# Breaking the cycle: unraveling the diagnostic, pathophysiological and treatment challenges of refractory migraine

**DOI:** 10.3389/fneur.2023.1263535

**Published:** 2023-09-27

**Authors:** Jennifer Robblee

**Affiliations:** Department of Neurology, Dignity Health, St Joseph’s Hospital and Medical Center, Lewis Headache Clinic, Barrow Neurological Institute, Phoenix, AZ, United States

**Keywords:** refractory migraine, intractable migraine, chronic migraine, chronic daily headache, resistant migraine, diagnosis, management

## Abstract

**Background:**

Refractory migraine is a poorly described complication of migraine in which migraine has chronified and become resistant to standard treatments. The true prevalence is unknown, but medication resistance is common in headache clinic patient populations. Given the lack of response to treatment, this patient population is extremely difficult to treat with limited guidance in the literature.

**Objective:**

To review the diagnostic, pathophysiological, and management challenges in the refractory migraine population.

**Discussion:**

There are no accepted, or even ICHD-3 appendix, diagnostic criteria for refractory migraine though several proposed criteria exist. Current proposed criteria often have low bars for refractoriness while also not meeting the needs of pediatrics, lower socioeconomic status, and developing nations. Pathophysiology is unknown but can be hypothesized as a persistent “on” state as a progression from chronic migraine with increasing central sensitization, but there may be heterogeneity in the underlying pathophysiology. No guidelines exist for treatment of refractory migraine; once all guideline-based treatments are tried, treatment consists of n-of-1 treatment trials paired with non-pharmacologic management.

**Conclusion:**

Refractory migraine is poorly described diagnostically, its pathophysiology can only be guessed at by extension of chronic migraine, and treatment is more the art than science of medicine. Navigating care of this refractory population will require multidisciplinary care models and an emphasis on future research to answer these unknowns.

## Introduction

Refractory migraine is poorly understood but likely represents migraine progression. These patients are resistant to guideline-based treatment, though the threshold for refractory is a matter of debate. Refractory is the most common term used though previous publications have used the term intractable and recently the European Headache Federation (EHF) proposed resistant migraine as a stage before refractory migraine (1–8).

Migraine is present in 14–15% of the population with a female preponderance (9). Chronic migraine has at least 15 headache days per month, of which 8 are migraine days, and represents 6.6–8.8% of patients with migraine (10). The proportion of patients refractory to treatment is unknown as no consistent diagnostic criteria have been accepted into the International Classification of Headache Disorders 3rd edition (ICHD-3) (11). Headache disorders are rated the second most disabling condition worldwide based on years lived with a disability (12). Those with refractory migraine are likely among the most disabled of the migraine population.

The purpose of this review article is to provide a comprehensive overview of the current knowledge and theories regarding refractory migraine. It will focus on the diagnosis, hypothesized pathophysiology, and management of refractory migraine. Additionally, the review will highlight the existing gaps in understanding and suggest future directions for research in this field.

## Diagnostic challenges

### Differentiating refractory migraine from other headache disorders

Refractory migraine is likely a subtype or progression from chronic migraine, though some argue that episodic migraine could be refractory depending on criteria used (13). The main headache disorder to differentiate from refractory migraine is medication overuse headache (MOH) (7).

Medication overuse itself does not exclude a diagnosis of refractory migraine, but MOH should be ruled out as a mimicker. MOH often presents as a chronic daily headache that can be refractory to both acute and preventive migraine treatments. It has long been known that MOH is major risk factor for conversion of episodic migraine into chronic migraine (14). There may be an increased risk of MOH in females, those with lower socioeconomic status, comorbid depression or anxiety, comorbid chronic pain disorders, and in the setting of cannabis use (15–17). MOH does not appear to be drug class specific, but rather occurs in predisposed patients with a primary headache disorder like migraine in the setting of medication overuse; however medication overuse does not automatically denote the disorder of MOH (11). To confirm the diagnosis of MOH, withdrawal of the causative medication(s) leading to significant improvement in headache is required (11). Hence if overused acute medication is withdrawn but no improvement occurs after a period of time, then MOH is unlikely. MOH relapses are more common in those with overuse of opioids, ergotamines, caffeine-containing medications, and combination medications (18). Those with MOH are less likely to respond to treatment hence can mimic refractory migraine, but some patients with MOH will improve with initiation of preventive treatments especially from migraine-specific medications like the calcitonin gene-related peptide (CGRP) monoclonal antibodies (19, 20). Some patients with MOH will spontaneously remit (21). Clinical trials have shown that various MOH management approaches work, but the best approach is to start a preventive treatment with or without planned medication withdrawal (22, 23). Patients unable to successfully withdraw overused acute treatments may need inpatient detoxification (24).

Beyond MOH, it is important to ensure secondary disorders like a cerebrospinal fluid (CSF) leak have been ruled out (25). There is also a possibility that some patients diagnosed with refractory migraine have underlying etiologies yet to be discovered, as evidenced by the case series of Nutcracker syndrome presenting with isolated chronic daily headache (26).

### Criteria for diagnosing refractory migraine

Multiple diagnostic criteria for refractory migraine have been proposed. The most recent diagnostic criteria are those proposed by the European Headache Federation (EHF) (8). They differentiate between resistant versus refractory migraine to provide two levels of severity with resistant migraine being debilitating despite trial of 3 drug classes while refractory migraine remains debilitating despite trying all drug classes. The authors noted that in many European countries, access to new drug classes like CGRP medications is already restricted to difficult to treat patients with one example provided that in Germany CGRP medications are restricted to those patients with episodic migraine who have tried 5 medications or with chronic migraine who have tried 6 medications including onabotulinumtoxinA.

#### Refractory migraine

- Established diagnosis of 1.1 Migraine without aura and/or 1.2 Migraine with aura or 1.3 Chronic migraine according to ICHD-III criteria.
- Debilitating headache for at least 8 days per month for at least 3 months.
- Failure and/or contraindication to all classes with established evidence for migraine prevention, given at an appropriate dose for an appropriate duration.

They define a debilitating headache impairing daily activity despite at least 2 ineffective triptan trials (8). Their recognized drug classes are antidepressants (amitriptyline and venlafaxine), antiepileptics (topiramate and valproate), beta blockers (atenolol, metoprolol, propranolol, timolol), calcium channel blockers (flunarizine or cinnarizine), CGRP medications (monoclonal antibodies or gepants), angiotensin pathway blockers (candesartan or lisinopril), onabotulinumtoxinA, and allowance for newly developed medications. Note that in the United States we do not have access to calcium channel blockers like flunarizine. Lack of tolerance and contraindications can count toward its failure in the EHF proposed criterion C.

Other refractory migraine criteria have been proposed including Goadsby et al. (1), American Headache Society (2), D’Amico et al. (3), Silberstein et al. (4), Austrian Consensus Group (5), European Headache Federation (6), as well as D’Antona and Matharu (7). See Table 1 for a comparison. Prior to then Valencia et al. (27) described poorly controlled primary headaches. Many groups are actively developing criteria as well. In the meantime, large variations exist in how refractory migraine is defined. Pharmaceutical trials define a “refractory” population as 2 to 4 prior preventives (28–31). However, this range does not mirror the reality of subspecialty clinics where our refractory patients may have tried 20 or higher without response.

**Table 1:** Evolution of the proposed criteria for refractory migraine.

First author	Goadsby	Schulman	D’Amico	Silberstein	Martelletti	Wober	D’Antona	Sacco	
Year	2006 (1)	2008 (2)	2008 (3)	2010 (4)	2014 (6)	2014 (5)	2019 (7)	2020 (8)	
Group	World Federation of Neurology meeting group	Refractory Headache group, American Headache Society	Independent group	Independent group	European Headache Federation	Austrian Consensus Group	Independent Group	European Headache Federation	
Terminology	Intractable Headache*	Refractory migraine**	Refractory chronic migraine	Intractable Headache	Refractory chronic migraine	Refractory chronic migraine	Refractory Migraine	Resistant migraine	Refractory migraine
MOH allowed?	Consider MO	Modifier	Yes	Yes	No	No	MO but not MOH	MO allowed	MO but not MOH
Preventives tried	4 classes	2 classes	All 1st-line^	Stratified by level^^	3 classes	3 classes	5 classes	3 classes	All^^^
Intolerance included as failure	Yes	Yes	Yes^	Yes	N/A	Yes	Yes	Yes	Yes
Contraindications included as failure	Yes	No***	Yes^	Yes	Yes	Yes	Yes	Yes	Yes
Acute meds tried	N/A	Yes	No	Stratified by level^^	No	No	No	Yes	Yes
Minimum headache days?	N/A	No	15	No	15	15	15	8	8
Disability required	Yes	Yes	Yes	Modifier	No	Yes	No	Yes	Yes
Workup needed?	N/A	N/A	Treat comorbidities	N/A	Yes	Yes	No	Consider DDx	Consider DDx

1st-line = first-line, DDX, Differential Diagnosis; MO, Medication overuse; MOH, Medication overuse headache; N/A, Not discussed. *Discussed intractable migraine as well as intractable cluster headache. **They differentiate refractory migraine and refractory chronic migraine. ***Discussed for acute medications but not for preventives. ^In addition to all first-line, they also recommend that some second and/or third-line agents have been tried. They also recommend that one medication from each class is insufficient. They also state that contraindicated and poorly tolerated medications should be avoided. ^^Triaging of preventive treatment severity can be summarized as Class I (mild) after 1 drug, Class II (moderate) after 2 drugs, Class III (Severe) after 3 drugs, and class IV (Very severe) are infusion/inpatient treatment. Triaging of acute treatment can be summarized as Class I is mild with lack of response to 2 NSAIDs and/or combination medications; Class II is moderate with additional lack of response to triptans/ergot; and Class III is severe with additional lack of response to parenteral treatment like opioids, antidopaminergics, and steroids. ^^^All evidence-based medication categories from a list of 7 categories with an 8th allowing for new treatments.

### Potential pitfalls

When diagnosing refractory migraine, the focus is on lack of response to preventive treatment. However, some criteria like EHF’s argue that lack of response to acute treatments needs consideration (8). I would argue that refractory migraine is a chronic disease state with resistance to preventive treatment. Response to acute treatment focuses on individual attacks and is more relevant to status migrainosus considerations (11).

Another pitfall is treatment access. The threshold for refractory migraine may look different in the Unites States versus in Africa where access to treatment like CGRP monoclonal antibodies is unlikely (32). Also consider pediatric versus adults with migraine; the threshold of refractoriness in an 8-year-old may need to be different than in a 45-year-old (33). Finally, even in the United States, the state of health insurance limits access to medication due to unaffordable copays (34). The threshold for refractoriness may need to be malleable enough to apply to country, age group, and socioeconomic group.

Another issue to consider is whether intolerance or contraindications count toward refractoriness. Refractory is defined as “resistant to treatment or cure.” (35) Sensitivity to multiple medications is common in a headache clinic, but that patient simply has many inadequate trials preventing evaluation of refractoriness. In fact, studies have demonstrated that clinical trials for migraine preventive treatment show nocebo rates of 42.78%, and one study looking at a specific nocebo of delayed headache after placebo infusion was 15.5% (36, 37). Relative versus absolute contraindications can also be an issue. For example, avoiding divalproex in a female of child-bearing age does not mean she would not respond to it. Sacco et al., in discussing resistant versus refractory migraine, specifically mention the hypothetical situation in which a patient has contraindications to all evidence-based classes (8). These situations may be appropriate for the ICHD-3’s use of “probable” diagnoses. True refractory migraine likely relies on ineffective treatment while probable refractory migraine may allow intolerance or contraindications. If using resistant migraine, the preferable use of the proposed diagnostic criteria would be 3 drug classes that are ineffective rather than relying on those not tried due to contraindications (8).

### The role of comorbidities

Co-morbid pain conditions are very common in migraine with one study reporting that 51% of patients with migraine have one or more concurrent pain condition(s) (38). That number increases past 70% in patients with chronic migraine (38). Fibromyalgia has been reported in 10–30% of patients with migraine with one study finding increased headache frequency to be predictive (38). One might expect even higher rates in those with refractory migraine, but this association has not been studied. There is a dose response relationship between allodynia and the number of comorbid pain conditions in migraine (39). Migraine is also associated with non-pain conditions including depression, anxiety, insomnia, psoriasis, allergy, diabetes, and asthma (40). More concurrent comorbidities is associated with increasing migraine attack frequency (40). Entities commonly seen in clinic include postural orthostatic tachycardia syndrome (POTS) and hypermobility; migraine is seen in one-third of POTS and in half of patients with hypermobility (41–44). While this dose response relationship has not been studied in refractory migraine, it could be predicted that a similar relationship would be seen.

## Pathophysiology of refractory migraine

### Current understanding of migraine mechanisms

Migraine is a complex sensory processing disorder with dysfunction of the trigeminovascular system including activation of trigeminal pathways, neurogenic inflammation, and release of neuropeptides like CGRP. Disease progression leads to increasing frequency of attacks and allodynia (45, 46). How this process progresses to refractory is unknown.

In looking at the mechanisms that explain progression from episodic to chronic migraine, the hypothalamus is often mentioned, it shows increased activation and connectivity to the spinal trigeminal nucleus in chronic migraine on functional magnetic resonance imaging (fMRI) (47). The hypothalamus is integral to migraine attack generation; hence one theory of refractory migraine pathophysiology is that enhanced hypothalamic activation perpetuates the active migraine state preventing treatment response (48). Progression is also associated with volume changes in regions of interests (ROIs), but the exact patterns still need to be elucidated (47). Over time there appears to be at least two broad underlying mechanisms for progression including prolonged nociceptive activity and neurogenic inflammation leading to hyperexcitability from sensitization as well as lack of habituation due to dysfunction of inhibitory brainstem pain control (47). The resulting central sensitization causes a brain state with increased spontaneous neural activity and reduced activation thresholds causing hypersensitivity to stimuli, reduced nocioceptive inhibition, and larger nocioceptive receptive fields (49).

Central sensitization represents hyperactivation of nociceptive pathways with dysfunction of thalamocortical modulation (45). Reduced functional connective on fMRI of both the default mode and executive networks has been associated with allodynia, a marker of central sensitization, without volumetric gray matter changes suggesting that functional changes precede any structural changes (50). fMRI has also shown evidence of hyperactivity of the spinal trigeminal nucleus and posterior thalamus with loss of descending pain inhibition (51). Allodynia predicts migraine chronification and is associated with longer disease duration, higher headache frequency, and worse outcomes (46, 52, 53). Treatment like triptans are most effective early in an attack prior to development of central sensitization (54). Allodynia also predicts lack of response to treatments like galcanezumab or onabotulinumtoxinA (55, 56). It follows that clinical and radiologic indicators of central sensitization should be predictive of refractory migraine, but further study is needed to assess that hypothesis.

### Risk factors for progression

Episodic migraine progresses to chronic migraine at a rate of 2% per year; the rate of progression to refractory migraine is unknown (57). Predictors of conversion from episodic to chronic migraine include cutaneous allodynia (45, 46), depression (58), MOH (59, 60), pain catastrophizing with a poor internal locus of control (61–63), lower socioeconomic status (64), and having multiple comorbidities (65). It is unknown if these risk factors also apply to refractory migraine however some studies have looked at predictors of response to specific treatments, which can be used as an indirect way to assess refractoriness in general. For instance, poor response to CGRP monoclonal antibodies is predicted by having prior ineffective treatments (66, 67). A different study on the use of erenumab in chronic migraine with concurrent MOH found that non-responders had 7.86 ± 1.85 prior ineffective treatments compared to 5.06 ± 1.62 in responders (*p* < 0.0001) (68). It should be noted that prior ineffective treatment does not negate the possibility of a response; a study from Germany showed that even with 5 or more prior ineffective treatments there was still at least a 50% response in 41.9% of patients with chronic migraine (69). In fact, even a lack of response to one CGRP monoclonal antibody does not negate response to a different one demonstrating the complexity in predicting treatment response (70). As a further complication to assessing refractoriness, relying on response at 3 month using a 50% responder rate may exclude approximately 16% of patient who would ultimately respond (71).

In looking at super-responders (75–100% responders) to a specific treatment category like CGRP monoclonal antibodies, studies demonstrate that they are more likely to have typical migraine features like unilateral pain, throbbing quality and vomiting; they also tended to have episodic migraine and a good triptan response (59). Studies looking at factors predicting at least a 50% response to CGRP monoclonal antibodies show that treatment responders are younger, have a lower headache frequency, unilateral pain ± unilateral allodynia but no interictal allodynia, unilateral cranial autonomic symptoms, more nausea/vomiting, more photophobia, lack of obesity, better response to triptans, less MOH, less pain catastrophizing, and less depression (67, 72–75). Conversely, a chronic daily headache at baseline is predictive of a poor response (67). MOH may not only be predictive of poor treatment response, but duration of MOH and the number of overused analgesia may also be predictive (68). One study found that cluster C personality disorders and significant life stressors predict poor response to erenumab (76). Based on a neuroimaging study, a lower baseline cerebral blood flow velocity in the middle cerebral arteries may predict a good response to CGRP monoclonal antibodies (77).

Poor treatment response to onabotulinumtoxinA has been associated with longer disease duration (78). There may be less of a decrease in headache days from onabotulinumtoxinA in those with allodynia, MOH and depression (56, 79). However a different study found improved response to onabotulinumtoxinA in patients with allodynia or pericranial muscle tenderness (80). One interesting study found that 74% of responders to onabotulinumtoxinA describe an imploding headache (i.e., external force sensation) and 13% described ocular pain while 92% of non-responders describe an exploding headache (internal pressure sensation) (81). Increased onabotulinumtoxinA response with ocular pain was also seen in a second study (82). At the biochemical level, certain plasma protein levels may predict response to onabotulinumtoxinA including CGRP, vasoactive intestinal peptide (VIP) and pentraxin 3 (PTX3) (79). A neuroimaging study found that iron deposition in the periaqueductal gray, a finding associated with chronic migraine as well as endothelial dysfunction and disrupted blood–brain barrier, was associated with worse onabotulinumtoxinA response (83, 84).

Based on the above findings, one could hypothesize that refractory migraine is more likely in older patients with longer disease duration who have bilateral imploding headache with less throbbing, pericranial muscle tenderness, triptan response and cranial autonomic symptoms, but more nausea/vomiting, allodynia, depression, stressors, and pain catastrophizing as well as higher rates of obesity, cluster C personality disorders and MOH. While not known, there may even be a dose response with more treatment failures corresponding to worse refractoriness. While we typically rely on assessment after 3 months of treatment, these patients may need longer trials and may benefit from trying another medication from a category previously tried. Further studies using biochemical and neuroimaging analysis are needed but some features may be predictive of refractory migraine like plasma protein levels of CGRP, blood flow velocities and the presence of iron deposition.

### Pharmacogenomics

Pharmacogenomics to predict treatment response in migraine is limited and not used clinically. Other than rare monogenic migraine like familial hemiplegic migraine, migraine is polygenetic with each gene having a small effect size but overall disease heritability of 35 to 60% (83). First degree relatives have a higher risk of migraine that increases with higher pain severity and attack frequency (83, 85). There are at least 180 loci associated with migraine (83, 86, 87). In a pharmacogenomics migraine study, verapamil-responders were compared to non-responders with 6 gene polymorphisms predictive of response. Polymorphisms of the 5-HT_1B_ receptor gene are associated with sumatriptan response (88). Otherwise, studies on genetics and refractoriness are absent.

### Neuroimaging insights

In a 2023 systematic review and meta-analysis, 40 migraine studies (*n* = 3297 patients) using voxel-based morphometry to compare migraine to healthy controls were assessed (89). Coordinate-based meta-analysis via 2 separate methodologies (anisotropic effect size-signed differential mapping and activation likelihood estimation) was used. Between these two methodologies, they found increased gray matter volume of the bilateral amygdala, bilateral parahippocampus, bilateral temporal poles, bilateral superior temporal gyri, left hippocampus, left middle temporal gyrus, right superior frontal gyrus but decreased volume of the left insula, bilateral cerebellum, right dorsal medulla, bilateral Rolandic operculum, right middle frontal gyrus, and right inferior parietal gyrus. The main finding found across both methodologies was gray matter increase in the left parahippocampus but decrease in the left insula. Broader variation in gray matter volumes were seen when subgroups like migraine with versus without aura or episodic versus chronic migraine were assessed. Further information on imaging findings in migraine is found when reviewing multivariate analysis for comparison to healthy controls or between migraine subgroups. In a 2016 study looking at structural and functional MRI findings using a multi-feature classification approach to compare migraine without aura (*n* = 21) to healthy control (*n* = 28), there was accuracy of 83.67% with sensitivity of 92.86% and specificity 71.43% (90). Discriminative structures include the anterior cingulate cortex, prefrontal cortex, orbitofrontal cortex and the insula (90). MRI can also differentiate episodic migraine from chronic migraine with 84.2% accuracy based on regional cortical thickness, cortical surface area, and volume (91). Further studies are needed to see if imaging can distinguish refractory migraine from chronic and episodic though at least one study demonstrated that treatment resistance is associated with more white matter hyperintensities (92).

## Management of patients with refractory migraine

### Evidence-based preventive treatment

The 2021 American Headache Society (AHS) Consensus Statement for the treatment of migraine is the most up to date guideline for the United States (93). The established preventive treatments from this consensus statement are erenumab, eptinezumab, fremanezumab, galcanezumab, onabotulinumtoxinA, candesartan, divalproex/valproate, propranolol, metoprolol, timolol, and topiramate. Amitriptyline, atenolol, lisinopril, memantine, nadolol, and venlafaxine are considered probably effective. Frovatriptan is an established peri-menstrual preventive treatment, and the guidelines advocate for neuromodulation devices. Nerve blocks are a standard treatments in many headache clinics but are not formally in the guidelines (94). Since the publication of this consensus statement, rimegepant and atogepant have been approved for the treatment of migraine (95). Beyond these options, small studies support the use of many other medications though many have conflicting or low quality evidence.

### Approach to pharmacologic management

The first step is to ensure the diagnosis is correct including ruling out MOH and that an adequate trial of evidence-based treatment was done. An adequate trial is 2 to 3 months at an adequate dose (93). The next appropriate step is to consider rational polypharmacy (96, 97). The AHS consensus statement advocates for the combination of a CGRP monoclonal antibody and onabotulinumtoxinA as a possibly effective therapy (93, 96). There is also increasing evidence that gepants can safely be used with CGRP monoclonal antibodies as another consideration for rational polypharmacy in patients with refractory migraine (98–102). The combination of a gepant and onabotulinumtoxinA has also been proposed as another example (103). While there are surprisingly few trials on combinations of the older non-specific treatments, there is some evidence for layering medications like topiramate and amitriptyline (104). The next step is the n-of-1 trial recognizing that by virtue of going outside guideline-based evidence these considerations have limited evidence (105).

### Ideas for n-of-1 trials

Preventive non-guideline treatments tried in migraine despite variable or limited evidence include anti-seizure medications like gabapentin, pregabalin, carbamazepine, oxcarbazepine, lamotrigine, levetiracetam and zonisamide (106, 107); calcium channel blockers like verapamil (108); anti-depressants like duloxetine, nortriptyline, doxepin, and phenelzine (109–111); atypical antipsychotic like olanzapine (112); and ergots like methylergonovine (113). Mirtazapine is an evidence-based treatment for tension-type headache, so could be tried for migraine (114). Acetazolamide is occasionally tried, especially in vestibular or hemiplegic migraine (115, 116). Amantadine, an NMDA receptor antagonist like memantine, has been tried in post-traumatic headache and migraine (117, 118). Observational studies suggest benefit from baclofen or tizanidine (119). Cannabinoids are often tried due to high public acceptance and there is good theoretical support for targeting the endocannabinoid system, however the risk of MOH has been raised (17, 120, 121). Despite the risk of MOH, occasionally daily triptans or NSAIDs are tried (122–124). Recently low-dose psilocybin has even been studied in a small cohort of patients with episodic migraine with more data available for the use of psychedelics in cluster headache (125, 126). Refractory migraine is a common indication for inpatient treatment using intravenous dihydroergotamine (DHE), ketamine, lidocaine, or propofol (127–130). In those responding to DHE, methylergonovine may be particularly of consideration (131). In those responding to lidocaine, mexiletine was often tried (132). A recent pilot study found that the ketogenic diet may be another consideration (133).

Opioids are occasionally considered despite low evidence, and may be initially started for non-cephalic pain (38, 134). However in headache medicine, opioids are a taboo due to MOH risk, especially if used greater than 9 days per month (11, 135). Opioid-related MOH represents only 4% of MOH-causing medications (136), but are high risk for central sensitization, MOH, progression from episodic to chronic migraine, increased healthcare utilization, worse disability and higher rates of mood disorders (137–139). Opioids are less effective than prochlorperazine, metoclopramide and dihydroergotamine when used acutely for migraine and may even impact treatment response (140–142). However, a limited group of patients with migraine do report improvement on opioids (143). Interesting the combination of NMDA receptor antagonism and opioids, like methadone or buprenorphine, may potentiate analgesia while reducing tolerance and hyperalgesia (144–146). Prospective cohort study has suggested that methadone, a racemic mixture of R and S-isomers as well as an NMDA receptor antagonist, may be beneficial daily at low doses for refractory chronic migraine (145). Further study is needed, but research into the use of delta opioid receptor agonists in migraine is also being looked into (147). Headache neurologists are hesitant to prescribe opioids due to these risks but there is a debate worth having of whether this group should have a trial of opioids, especially methadone or buprenorphine, with careful monitoring for the development of MOH over a pre-defined period like 3 months.

In patients with refractory migraine, surgical options, like invasive occipital nerve stimulation (ONS), may be considered. ONS has possible support from systematic review and meta-analyzes (17, 148, 149), however the studies are prone to bias due to small size and difficulty blinding with safety concerns including lead migration, infection and pain (150, 151). Long-term studies on ONS are limited in migraine, with the majority done in cluster headache, but persistence of benefit is reported (152, 153). Intuitively, response to occipital nerve block should predict ONS response but thus far it does not (154, 155). Some centers add supraorbital stimulation to ONS for better response though at least one study suggests that response is not sustained (156–158). Deep brain stimulation has been used in headache disorders, but there is no evidence for its use in migraine (159, 160). Finally, occipital nerve decompression is reported as a potentially effective treatment for some patients (161, 162).

### Multidisciplinary treatment plans

Beyond pharmacologic management, these patients require non-pharmacologic and multidisciplinary care. Behavioral treatments are highly recommended given cognitive constructs like pain catastrophizing, avoidance, and cephalalgiaphobia (163–165). Biofeedback and cognitive behavioral therapy (CBT) are mainstays of treatment often combined with techniques like mindfulness and relaxation therapy (166, 167). These treatments may be combined with pharmacologic treatments. For instance, CBT and amitriptyline have been shown to be synergistic (168, 169). Treatments like physical therapy, manual therapy, acupuncture, dry needling, and exercise are often used (170–174). In fact, exercise may have a synergistic benefit when used in combination with amitriptyline (175). Beyond strength training or aerobic exercise, yoga also has evidence for use (176). Finally other lifestyle interventions may be tried like trigger elimination, diet alterations, hydration, and sleep optimization (177, 178). Patients with refractory migraine will likely need a combination of these treatments.

### Preventing refractory migraine

At this time, we do not know how to prevent refractory migraine. Even for conversion from episodic to chronic migraine, there is conflicting evidence on the importance of starting preventive treatment (179, 180). For instance, studies have shown that the use of topiramate in patients with episodic migraine may prevent progression based on pooled results across 3 studies in which 2.1% (8/384) of patients on topiramate (100 mg) progressed to chronic migraine, while 4.3% (16/372) in the placebo group progressed over 26 weeks (179). Comparatively, the INTREPID study looked at topiramate (100 mg) in patients with high frequency episodic migraine and found no significant difference in the rate of conversion to chronic daily headache at 6 months when compared to the placebo group (180). There is also evidence that optimizing acute treatment of migraine may help prevent progression to chronic migraine (181). NSAIDs may even have a protective effect for those with less than 10–14 headache days per month (57). Beyond treating early with pharmacologic management, treatment of modifiable risk factors for progression like managing comorbidities and avoiding medication overuse may help prevent progression to at least chronic migraine (182, 183). Whether this data on preventing conversion from episodic to chronic migraine is relevant to preventing refractory migraine is unknown.

## Knowledge gaps and future research

The top research priority for refractory migraine is the development and acceptance of ICHD diagnostic criteria. Without a standard guide for diagnosis, all studies on epidemiology, pathophysiology, and treatment will not use a homogenous population. Once diagnostic criteria are accepted, research can be undertaken to clarify disease burden, which is likely high and represents a substantial proportion of subspecialty headache clinic patients. Pathophysiology can then be investigated using genetic studies, risk factor analysis, neuroimaging, and biochemical analysis. Once we clarify who we are treating (diagnosis) and what we are treating (pathophysiology) then studies can identify rational targets for therapy allowing for randomized controlled trials and ultimately guideline development. Research may even allow identification of these patients prior to becoming refractory, allowing early intervention to prevent this disease state or avoid years of ineffective treatment trials. This future state is a long way off, and the headache community cannot advocate enough for first pinning down accepted diagnostic criteria.

## Conclusion

Refractory migraine, representing the most debilitated and complex migraine population, has been largely overlooked. The urgent need for established diagnostic criteria is paramount to advancing research on pathophysiology and developing effective treatments. Currently, there are multiple proposed criteria without an official diagnosis in the ICHD3. Pathophysiology can only be hypothesized, and treatment approaches vary widely with reliance on low quality evidence driving n-of-1 treatment trials. Management of refractory migraine requires the art of medicine while awaiting scientific advancements.

## Author contributions

JR: Conceptualization, Data curation, Resources, Writing – original draft, Writing – review & editing.
